# Sterilization of paper during crisis

**DOI:** 10.1186/s13568-022-01345-6

**Published:** 2022-02-08

**Authors:** Fwzah H. Alshammari, Hebat-Allah A. Hussein

**Affiliations:** 1 Physical Department, University College of Nairiyah, University of Hafr Al Batin (UHB), Nairiyah, 31991 Saudi Arabia; 2 Biology Department, University College of Nairiyah, University of Hafr Al Batin (UHB), Nairiyah, 31991 Saudi Arabia; 3 Botany and Microbiology Department, Faculty of Science (Girls Branch), Al Azhar University, Cairo, 11751 Egypt

**Keywords:** Gamma radiation, Dry heating, Respiratory pathogen, Paper structure, *Escherichia coli*, *Salmonella typhi*

## Abstract

Paper sheets represent one of the infection risk sources inside educational and administrative institutions under biological pandemics. So, the present study aimed to validate the efficiency of gamma radiation or dry heat techniques to sterilize contaminated paper sheets with different indicator pathogens while retaining their structure. The results showed that gamma radiation at 6, 12, or 24 kGy can successfully kill Gram-positive bacteria such as *Bacillus cereus* and *Staphylococcus aureus*, Gram-negative bacteria such as *Escherichia coli* and *Salmonella typhi, *and fungi such as *Candida albicans*. Moreover, dry heating at 100 °C for 60 min, 150 °C for 30 min, or 200 °C for 15 min can be successful in paper decontamination of all tested species. Surprisingly, scanning electron microscopy (SEM) micrographs proved that gamma radiation at 6 kGy, dry heat at 100 °C for 60 min or 150 °C for 30 min or 200 °C for 15 min, is suitable for paper sheet sterilization while maintaining their structure. Ultimately, dry heat is a simple, effective, fast, safe, and inexpensive technique for paper sterilization. It may be used as a precautionary step inside educational institutions, especially during written examination periods, to ensure a safe life for academic members during biological pandemics such as COVID-19.

## Key points

SEM was used to investigate the structure of the sterilized paper.

Using dry heating is easy and safer than gamma radiation in paper sterilization.

Ovens can be available in all institutions.

## Introduction

SARS-CoV-2 is the virus that causes COVID-19 disease and its variants Alpha, Beta, Gamma, Delta, and Omicron, affect the educational process around the world. Many schools and universities became increasingly closed because the coronaviruses caused severe risks of death in some cases. Coronaviruses can remain infectious on surfaces for up to nine days at room temperature (Henwood 2020). When schools and universities reopen, paper sheets represent one of the infection risk sources in the academic community.

**Fig. 1:**
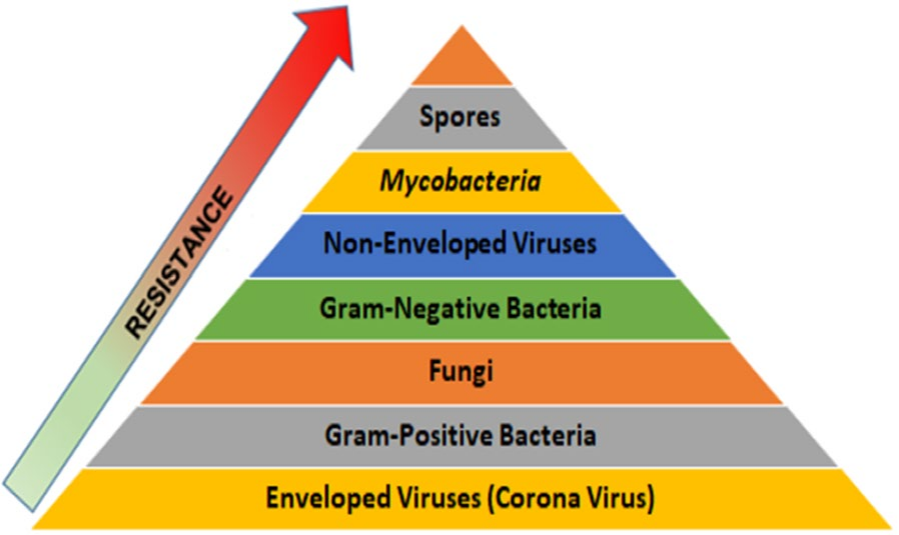
Resistance of microorganisms to sterilization (William and Weber 2008)

Sterilization is a process that effectively eliminates all pathogens, such as viruses, bacteria, fungi, and spore forms. Microorganisms vary widely in their resistance to disinfection. Bacterial spores have innate immunity. According to the relative scale of resistance (Fig. 1), coronaviruses are the most sensitive to disinfection (William and Weber 2008). Enveloped viruses, under the influence of dry heating or gamma radiation, are more susceptible to inactivation than viruses without envelopes. The lipids building the envelope undergo peroxidation (Blázquez et al. 2019). Therefore, the dose of radiation eliminating bacterial spores will also be destructive to enveloped viruses.

The utilization of biological indicators is represented as the most reliable, easy, and fast technique of sterilization control. These indicators are mentioned in the EN ISO 11138–1: 2017 standard concerning the sterilization of products (BSI 2017). The bio-indicator systems contain the spores of a nonpathogenic microorganism. They are suitable for a tested type with the highest resistance to a distinct sterilizing agent (Kierat et al. 2020).

On the other hand, the different sterilization techniques depend on the material type (Singh et al. 2016). Paper sterilization is an essential step for the preservation of books in libraries and plant specimens in herbaria. The antiseptic solution and spray, autoclave, or microwave sterilization are not suitable for paper disinfection (Li et al. 2020). Sterilization methods using Ultraviolet (UV) radiation are harmful to human eyesight and can also generate ozone. Moreover, UV sterilization of paper must be achieved one at a time because UV rays cannot penetrate more than one sheet. This technique is not effective in sterilizing a large number of paper sheets. In addition, gas methods leave an unpleasant odor, and it is also possible for gas to penetrate the material and release it slowly thereafter. Now, under the COVID-19 pandemic, it is necessary to find a proper technique for paper sterilization to protect the academic community from probable infections.

Gamma sterilization is a cold sterilization technique for microbial inactivation of different materials (Ali et al. 2016). International standards for radiation sterilization ask for evidence that a minimum dose of 25 kGy induces irreversible structural changes in many materials (Karina et al. 2018). Radiation sources such as Co60 and Cs137 can release high-energy electromagnetic gamma rays and effectively eliminate contaminating microorganisms (Silindir and Özer 2009). There are different opinions about the radiation dose to achieve the sterilization of paper sheets without damage. Gonzales et al. (2002) used 14.4 kGy to commercial papers and reported that their resistance remained with no change (Gonzalez et al. 2002). Gamma radiation from 3 to 15 kGy showed no significant effects on the properties of irradiated paper (D’Almeida et al. 2009). However, gamma rays are very effective in penetrating and sterilizing paper, and the lack of gamma cells in educational institutes makes this method unsuitable for paper sterilization.

In general, methods of paper sterilization have some challenges. The first challenge is maintaining the physical properties of paper samples. The second challenge is effectively decontaminating methods for different types of resistant pathogens. Dry heat sterilization represents the oldest technique used in sterilization. Interestingly, it is a safe and effective technique that can sterilize 2000 paper samples at a time while maintaining their structural properties. Additionally, oven equipment is suitable for the educational institutes that making this method the best choice for paper sterilization.

The actual sterilization time reaches an hour at a temperature of 160 °C to 170 °C (Rashed et al. 2020). Moreover, incubation at 50 °C or 60 °C for 30 min could inactivate viruses (Bertrand et al. 2012). A higher temperature is a safer option for the inactivation of SARS-CoV-2 (Xiang et al. 2020). Heat at 70 °C could kill a wide range of pathogens (Vieira and Pecchia 2018; Xiang et al. 2020). Many coronaviruses are inactive after exposure to temperatures: 90 min at 56 °C, 60 min at 67 °C, and 30 min at 75 °C (Duan et al. 2003). Concerning the physical properties of heat sterilized paper, using 175 °C improves the mechanical properties of the printing paper (Koubaa and Koran 2018).

The current study aimed to determine the minimum exposure time, heat, or radiation dose to sterilize paper sheets while maintaining its structural properties and the maximum exposure time, heat, or radiation dose that may cause paper damage.

## Materials and methods

### Contamination of paper

For contamination of paper samples, the reference strains used were as follows: Gram-positive bacteria such as *Bacillus cereus* ATCC-12,228 and *Staphylococcus aureus* ATCC-47077. Gram-negative bacteria such as *Escherichia coli* ATCC-25,922 and *Salmonella typhi* ATCC-15566. Fungi like *Candida albicans* ATCC-10,231. Cultures of the tested pathogens were prepared in nutrient broth medium overnight. The solutions of 10^4^ CFU/ml of the tested strains were done in sterilized tap water using a hemocytometer slide (Abdelhameed et al. 2019; Abdel-Monem et al. 2020).

After preparation of the microbial culture solution, printing paper (3 * 3 cm) samples were treated with the prepared solutions using sterilized swaps. The treated paper sections were exposed to radiation or temperature treatments.

### Sterilization methods

**Fig. 2:**
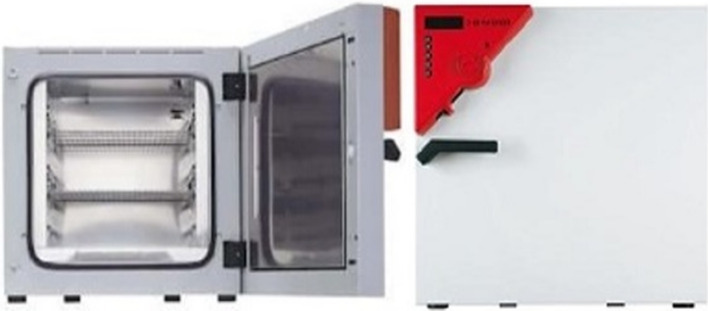
The Binder FED 53-UL Forced Convection Drying Oven electronically controlled APT.line™ preheating chamber

Paper sheets were sterilized by two different methods: gamma irradiation and dry heat. Furthermore, the change in the structural properties was evaluated. In the first method, paper sheets (3* 3 mm) were packed in Petri dishes and exposed to a gamma dose of 6, 12, or 24 kGy. The applied radiation was at a dose rate of 125 Gy/min using Canadian Gamma Cell 40- Cesium 137 biological sources at the National Center for Radiation Research and Technology (NCRRT), Cairo, Egypt. The dose rate level for paper samples (6, 12, and 24 kGy) at a dose of 0.6 Gy/sec. In the second method, contaminated paper sheets were exposed to high temperatures ranging from 100 °C, 150 °C, or 200 °C for different periods (15, 30, or 60 min) at the University College of Nairiyah, University of Hafr Al Batin, Saudi Arabia. Temperature treatments were applied using the Binder FED 53-UL Forced Convection Drying Oven electronically controlled APT.line™ preheating chamber (Fig. 2). The temperature ranges from 5 °C (32°F) above ambient temperature up to 300 °C (572 °F). There is a DS controller with an integrated timer of 0 to 99 h and a digital temperature setting with an accuracy of one degree. Independently adjustable temperature safety device class 2 (DIN 12,880), with visual temperature alarm. There is also an adjustable front ventilation flap slide and rear exhaust ø 50 mm (1.97 inches).

### Microbial counts determination

The treated paper samples were divided into two groups: the first group was put into 10 ml sterilized water and vortexed. From serial dilutions, 1 ml was inoculated on the surface of nutrient agar plates. After incubation, the number of grown colonies was counted and recorded. The other group was placed onto nutrient agar plate surfaces and incubated for 48 h at 37 °C, and the growth of microbes was noted (Mourad et al. 2019; Abdel-Monem et al. 2020).

### Scanning electron microscope (SEM)

Scanning electron microscopy- energy dispersive X-ray spectrometry (SEM–EDX). Backscattered electron images in the SEM display compositional contrast that results from different atomic number elements and their distribution. Energy Dispersive X-ray Spectroscopy (EDX) allows one to identify the structure of materials. The paper samples were analyzed on double-sided tape on aluminum stubs. The morphology of the sterilized paper was detected through scanning electron microscopy (SEM) using a field-emission scanning electron microscope (Model, Quanta 250 FEG; field-emission Gun, JEM2100, Jeol, Japan) (Mourad et al. 2019).

## Results

### Gamma radiation sterilization

**Fig. 3:**
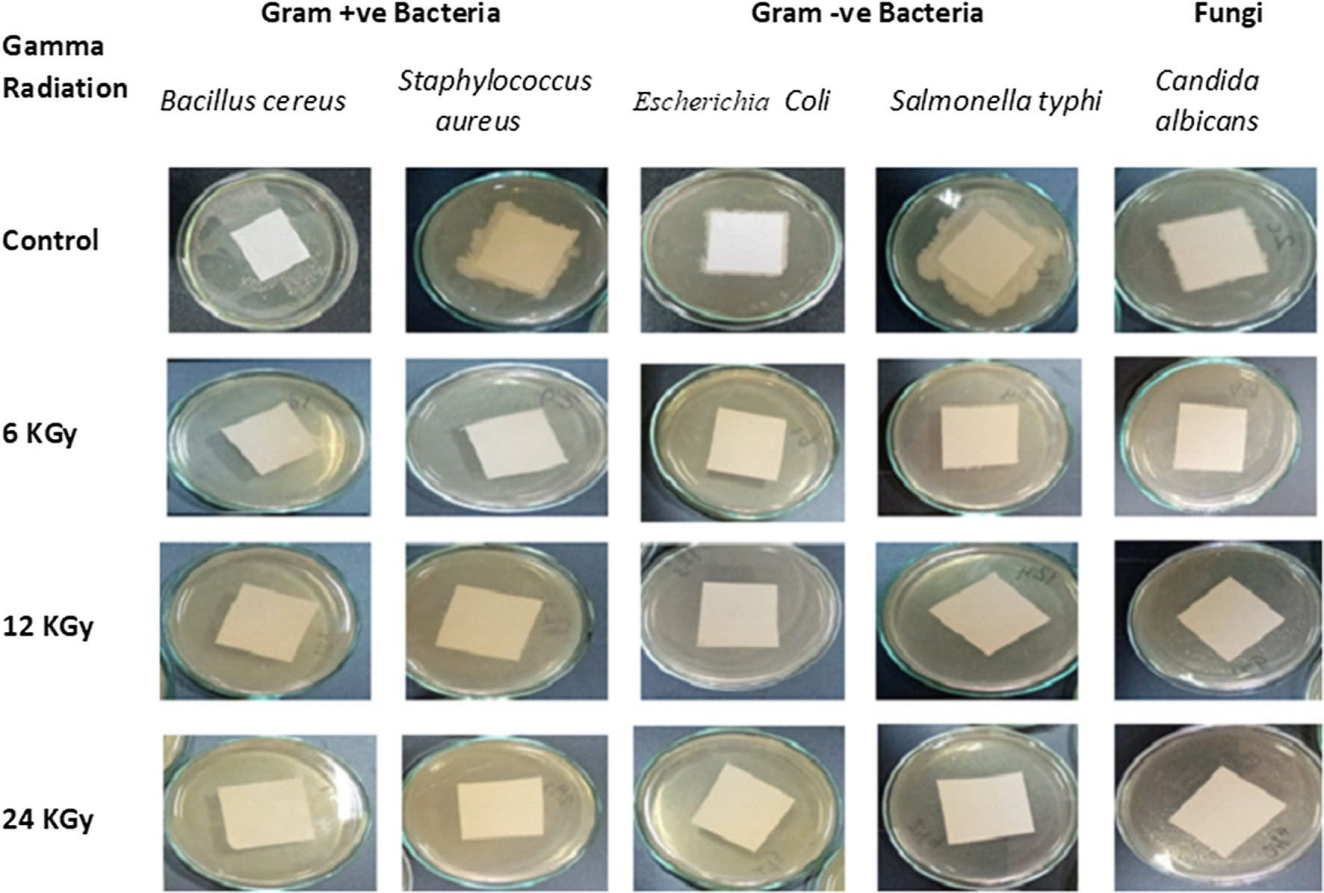
The efficiency of gamma radiation in the sterilization of pathogenic contaminated paper

The data in Table 1 and Fig. 3 show that γ-radiation at 6, 12, or 24 kGy is an effective technique in the sterilization of paper sheets contaminated with Gram-positive bacteria such as *Bacillus cereus* and *Staphylococcus aureus*, Gram-negative bacteria such as *Escherichia coli* and *Salmonella typhi, *and fungi such as *Candida albicans*.

**Table 1:** The efficiency of gamma radiation in the sterilization of pathogenic contaminated paper

Treatments		Average no. of organisms				Inactivation of test organism %
Tested organism	γ-radiation	Control	6 kGy	12 kGy	24 kGy	
Gram-positive bacteria	*Bacillus cereus*	10 × 10^6^	0.0	0.0	0.0	100
	*Staphylococcus aureus*	8 × 10^7^	0.0	0.0	0.0	100
Gram-negative bacteria	*Escherichia coli*	9 × 10^6^	0.0	0.0	0.0	100
	*Salmonella typhi*	12 × 10^6^	0.0	0.0	0.0	100
Fungi	*Candida albicans*	8 × 10^6^	0.0	0.0	0.0	100

### Dry heating sterilization

**Fig. 4:**
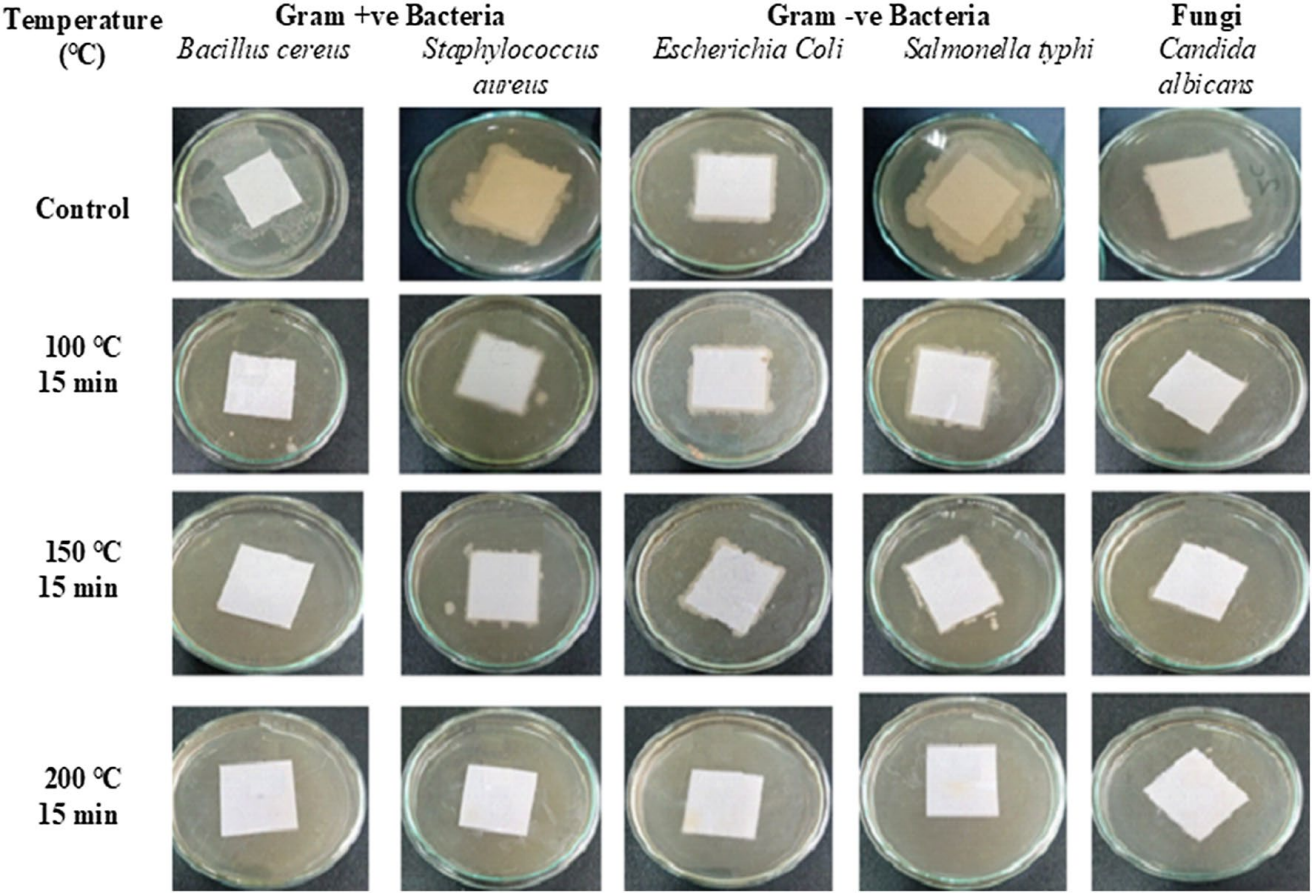
The efficiency of dry heating for 15 min in the sterilization of pathogenic contaminated paper

The effectiveness of dry heating in the sterilization of paper samples depends on the type of tested pathogenic species, temperature degree, and exposure period. Dry heating sterilization at 100 °C, 150 °C, and 200 °C for 15 min markedly reduced the number of *Bacillus cereus* by 99.0%, 99.9%, and 100%, *Staphylococcus aureus *by 99.0%, 99.6%, and 100%, *E. coli* by 97.2%, 97.7%, 100%*, Salmonella typhi* 98.3%, 98.6% and 100% and *Candida albicans* by 97.1%, 97.6%, and 100%, respectively, compared to the corresponding control (Table 2 and Fig. 4).

**Table 2:** The efficiency of dry heat at different periods in the sterilization of pathogenic contaminated paper

Treatments			Average no. of organisms				Inactivation of tested organism %		
Time (min)	Tested organism	Temperature °C	Control	100 °C	150 °C	200 °C	100 °C	150 °C	200 °C
15 min	Gram-positive bacteria	*Bacillus cereus*	10 × 10^6^	6 × 10^3^	5 × 10^3^	0.0	99.0	99.9	100
		*Staphylococcus aureus*	8 × 10^7^	30 × 10^4^	27 × 10^4^	0.0	99.0	99.6	100
	Gram-negative bacteria	*Escherichia coli*	9 × 10^6^	25 × 10^4^	21 × 10^4^	0.0	97.2	97.7	100
		*Salmonella typhi*	12 × 10^6^	20 × 10^4^	17 × 10^4^	0.0	98.3	98.6	100
	Fungi	*Candida albicans*	8 × 10^6^	23 × 10^4^	19 × 10^4^	0.0	97.1	97.6	100
30 min	Gram-positive bacteria	*Bacillus cereus*	10 × 10^6^	0.0	0.0	0.0	100	100	100
		*Staphylococcus aureus*	8 × 10^7^	7 × 10^2^	0.0	0.0	99.9	100	100
	Gram-negative bacteria	*Escherichia coli*	9 × 10^6^	5 × 10^2^	0.0	0.0	99.9	100	100
		*Salmonella typhi*	12 × 10^6^	3.5 × 10^2^	0.0	0.0	99.9	100	100
	Fungi	*Candida albicans*	8 × 10^6^	4 × 10^2^	0.0	0.0	99.9	100	100
60 min	Gram-positive bacteria	*Bacillus cereus*	10 × 10^6^	0.0	0.0	0.0	100	100	100
		*Staphylococcus aureus*	8 × 10^7^	0.0	0.0	0.0	100	100	100
	Gram-negative bacteria	*Escherichia coli*	9 × 10^6^	0.0	0.0	0.0	100	100	100
		*Salmonella typhi*	12 × 10^6^	0.0	0.0	0.0	100	100	100
	Fungi	*Candida albicans*	8 × 10^6^	0.0	0.0	0.0	100	100	100

**Fig. 5:**
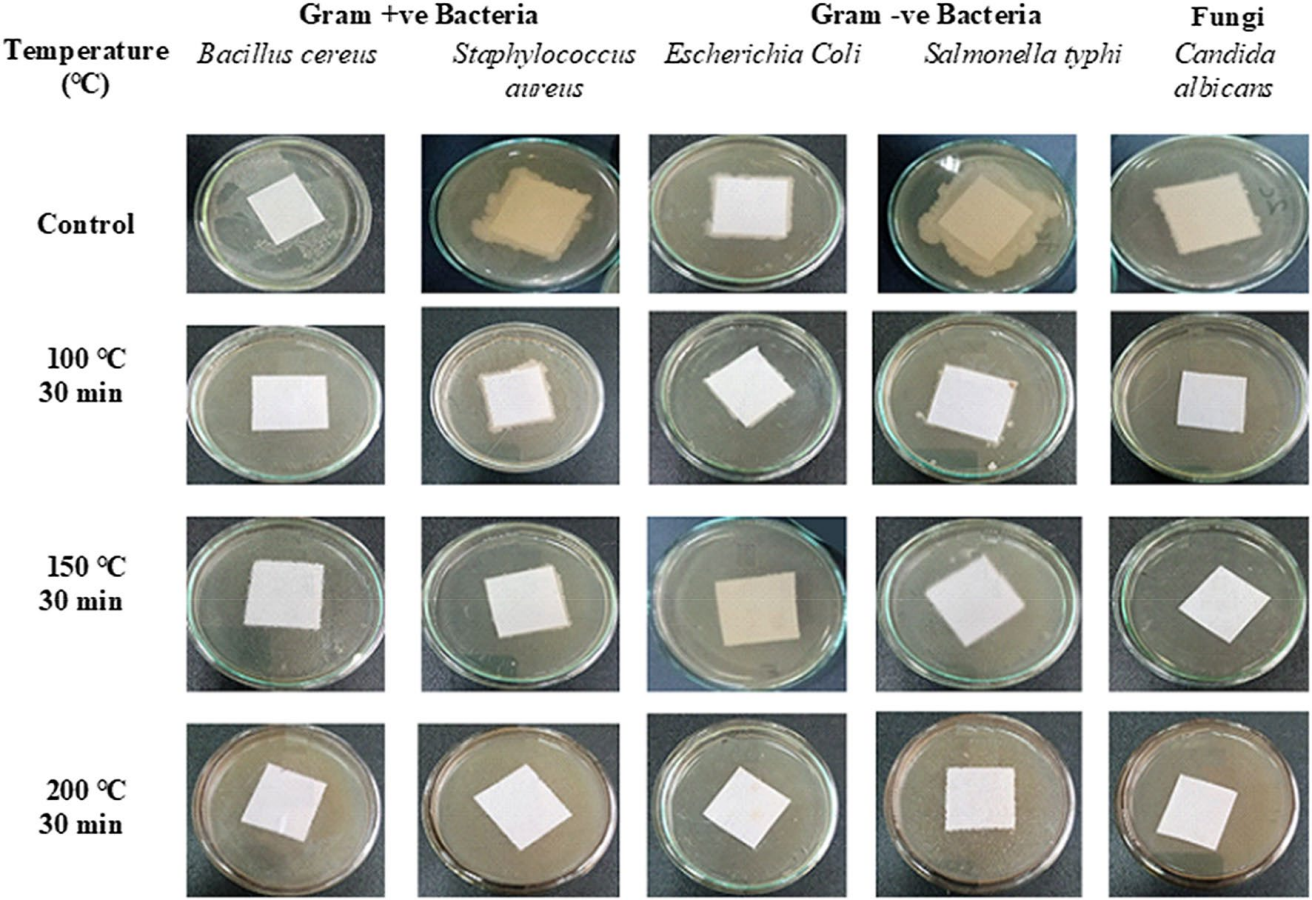
The efficiency of dry heating for 30 min in the sterilization of pathogenic contaminated paper

The data presented in Table 2 and Fig. 5 show that temperature at 100 °C for 30 min inhibited the growth of *Bacillus cereus* by 100% and the number of *Staphylococcus aureus, E. coli, Salmonella typhi, *and *Candida albicans* by 99.9% for each mentioned pathogen compared to the corresponding controls. Moreover, 150 °C or 200 °C for 30 min can destroy all tested pathogenic microorganism-contaminated paper sheets. In addition, dry sterilization at 100 °C, 150 °C, or 200 °C for one hour is an effective method for killing all tested pathogens.

### Effect of gamma radiation on paper structure

**Fig. 6:**
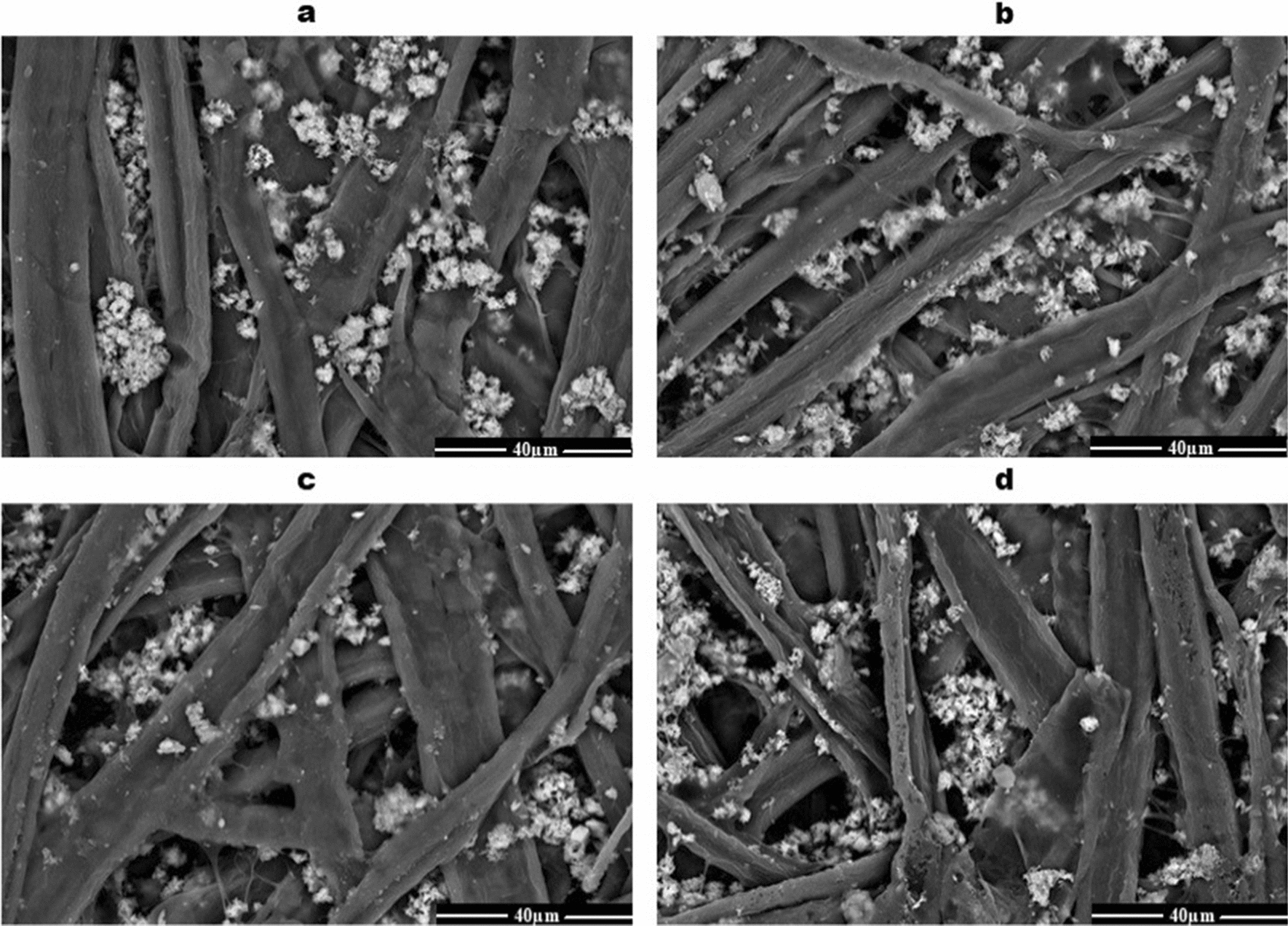
SEM micrograph of the paper structure after exposure to the gamma radiation doses. Control (**a**), 6 kGy (**b**), 12 kGy (**c**), and 24 kGy (**d**)

Scanning electron microscopy **(**SEM) was used to study the structure and morphology of the sterilized paper sheets. Each SEM micrograph in Fig. 6 shows the changes in the paper structure after gamma sterilization. Control samples of paper sheets have a high density of intertwined cellulose fibers, different shapes, and sizes, and calcium carbonate agglomerates (Fig. 6a). On the other hand, gamma radiation at 6 kGy caused the flatness of the cellulose microfibrils, providing a larger surface area (Fig. 6b). In addition, a high-density structure of intertwined cellulose fibers and calcium carbonate agglomerates was observed. However, the irradiated paper sheets with 12 kGy showed a decline in binding joints, resulting in a lack of interfiber forces (Fig. 6c). Furthermore, the high dose of γ-radiation (24 kGy) resulted in a severe degree of hornification (Fig. 6d).

### Effect of dry heat sterilization on paper structure

**Fig. 7:**
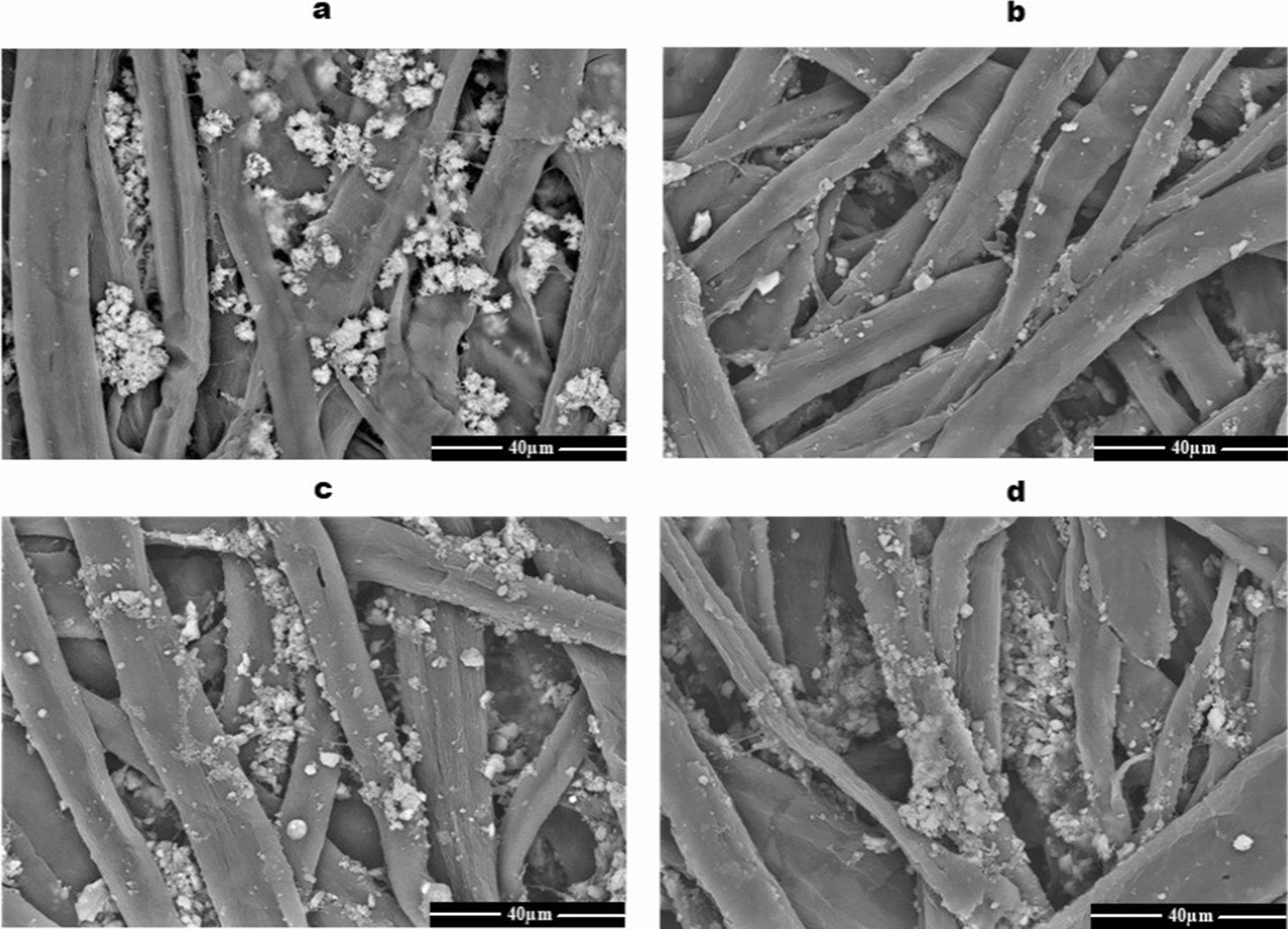
SEM micrograph of the paper structure after exposure to the dry heat treatments. Control (**a**), 100 °C (**b**), 150 °C (**c**), and 200 °C (**d**)

In Fig. 7, SEM images of the dry heating paper sheets for one hour were examined and compared to unheated sheets (Fig. 7a). The results showed that the sheet treated at 100 °C attained a slight reduction in bonding between cellulose microfibers (Fig. 7b). However, the deformation of cellulose microfibers was detected in the microstructure images of the dry heating paper sheets at 150 °C or 200 °C (Fig. 7c, d). Dry heated sheets at 200 °C gained a severe heterogeneous microstructure and a yellow color.

## Discussion

There are different sterilization techniques for the elimination of viruses depending on the material type, the tested pathogenic species, type of treatment, and exposure period. The present study investigates only gamma radiation techniques as cold sterilization and dry heat technique as heating sterilization. In the current work, γ-radiation represents an effective technique to sterilize pathogenic contaminated paper sheets. These results may be due to gamma radiation generating free radicals that react with biological molecules. DNA is highly susceptible to the effects of radiation (Kuefner et al. 2015). Damage to DNA molecules eventually leads to cell death (Sage and Shikazono 2017; Harrell et al. 2018).

In addition, the results indicate that dry heat can kill a wide range of pathogens. Dry heating sterilization is the best method for paper sterilization. The main reason is its low penetration, which retained the physical properties of paper. Moreover, oven equipment is convenient and economical (Xiang et al. 2020). On the other hand, wet sterilization could not be used for paper disinfection because of its high penetration. The high temperature and steam together may affect the paper structure more than dry heat only (Li et al. 2020). So, the potential of different methods should be investigated in future experiments.

Scanning electron microscopy (SEM) was used to investigate the structure and morphology of the dry heat and gamma-radiation sterilized paper sheets. Gamma radiation resulted in the flatness of the cellulose microfibrils. Additionally, it caused a high-density structure of intertwined cellulose fibers and calcium carbonate agglomerates. Moreover, it decreased the binding joints, resulting in the reduction of interfiber forces or a severe degree of hornification. These results may be due to a lack of the water-holding potential of cellulose microfibrils or dry conditions leading to lower swelling of the microfibres, density, and strength properties of paper sheets (Salmén and Stevanic 2018). Furthermore, the strength properties of cellulose fibers were due to the increased density of molecular cross-linking, depending on the gamma-ray dose. After 10 kGy, the strength properties of cellulose fibers decreased gradually with the γ-ray dose (Hoque et al. 2017).

The paper sheet treated at 100 °C for one hour attained a slight reduction in bonding between cellulose microfibrils. However, the deformation of cellulose microfibers was detected in the microstructure images of the dry heating paper sheets at 200 °C. The dry heated sheets at high temperatures gained a severe heterogeneous microstructure as well as a yellow color. These changes may be due to the paper components are susceptible to dryness conditions and losses of the swelling ability of microfibrils. When cellulose microfibers were dried in contact, they essentially healed together and became one (Hubbe 2014).

In conclusion, during a biological crisis like COVID- 19 pandemic, the risk of probably contaminated paper inside many institutions must be considered, especially with the reopening of schools, libraries, and universities. The present study proved that exposure to gamma radiation at 6 kGy or dry heat at 100℃ for 60 min can ensure the sterilization of paper while maintaining its physical and structural properties. Moreover, dry heat is a simple, effective, fast, safe, and inexpensive technique for paper sheet disinfections. Interestingly, it is the oldest and the best for paper sterilization. Ultimately, paper sterilization is an argent strategy to protect individuals from probable infection under a biological pandemic like COVID-19.

## Data Availability

Data are available upon request.

## References

[CR1] Abdel-Monem RA, Khalil AM, Darwesh OM, Hashim AI, Rabie ST (2020). Antibacterial properties of carboxymethyl chitosan Schiff-base nanocomposites loaded with silver nanoparticles. J Macromol Sci Part A Pure Appl Chem.

[CR2] Abdelhameed RM, Darwesh OM, Rocha J, Silva AMS (2019). IRMOF-3 biological activity enhancement by post-synthetic modification. Eur J Inorg Chem.

[CR3] Ali SI, Mohamed AA, Sameeh MY, Darwesh OM, Abd El-Razik TM (2016). Gamma-irradiation affects volatile oil constituents, fatty acid composition and antimicrobial activity of fennel (Foeniculum vulgare) seeds extract. Res J Pharm Biol Chem Sci.

[CR4] Bertrand I, Schijven JF, Sánchez G, Wyn-Jones P, Ottoson J, Morin T, Muscillo M, Verani M, Nasser A, de Roda Husman AM, Myrmel M, Sellwood J, Cook N, Gantzer C (2012). The impact of temperature on the inactivation of enteric viruses in food and water: A review. J Appl Microbiol.

[CR5] Blázquez E, Rodríguez C, Ródenas J, Navarro N, Riquelme C, Rosell R, Campbell J, Crenshaw J, Segalés J, Joan P, Polo J (2019). Evaluation of the effectiveness of the surepure turbulator ultraviolet-C irradiation equipment on inactivation of different enveloped and non-enveloped viruses inoculated in commercially collected liquid animal plasma. PLoS ONE.

[CR6] BSI (2017) BS EN ISO 11138–1:2017 Sterilization of health care products - Biological indicators Part 1: General requirements

[CR7] D’Almeida MLO, de Barbosa P, SM, Boaratti MFG, Borrely SI, (2009). Radiation effects on the integrity of paper. Radiat Phys Chem.

[CR8] Duan SM, Zhao XS, Wen RF, Huang JJ, Pi GH, Zhang SX, Han J, Bi SL, Ruan L, Dong XP (2003). Stability of SARS Coronavirus in Human Specimens and Environment and Its Sensitivity to Heating and UV Irradiation. Biomed Environ Sci.

[CR9] Gonzalez ME, Calvo AM, Kairiyama E (2002). Gamma radiation for preservation of biologically damaged paper. Radiat Phys Chem.

[CR10] Harrell CR, Djonov V, Fellabaum C, Volarevic V (2018). Risks of using sterilization by gamma radiation: The other side of the coin. Int J Med Sci.

[CR11] Henwood AF (2020). Coronavirus Disinfection in Histopathology J Histotechnol.

[CR12] Hoque MA, Bhuiya MAK, Saiduzzaman M, Islam MA, Khan MA (2017). Effect of γ (Gamma)-radiation on mechanical properties of raw and polyethylene glycol-modified bleached jute reinforced polyester composite. World J Eng.

[CR13] Hubbe MA (2014). Prospects for maintaining strength of paper and paperboard products while using less forest resources: A review. BioResources.

[CR14] Karina KM, Napolitano CM, Borrely SI (2018). Gamma radiation effects in packaging for sterilization of health products and their constituents paper and plastic film. Radiat Phys Chem.

[CR15] Kierat W, Augustyn W, Koper P, Pawlyta M, Chrusciel A, Wyrwol B (2020). The use of UVC irradiation to sterilize filtering facepiece masks limiting airborne cross-infection. Int J Environ Res Public Health.

[CR16] Koubaa A, Koran Z (2018) Effect of Press-Drying Parameters on Paper Properties. In: Pulp and Paper Processing. New York: InTech

[CR17] Kuefner MA, Brand M, Engert C, Schwab SA, Uder M (2015). Radiation Induced DNA Double-Strand Breaks in Radiology. RoFo Fortschritte Auf Dem Gebiet Der Rontgenstrahlen Und Der Bildgeb Verfahren.

[CR18] Li DF, Cadnum JL, Redmond SN, Jones LD, Pearlmutter B, Haq MF, Donskey CJ (2020). Steam treatment for rapid decontamination of N95 respirators and medical face masks. Am J Infect Control.

[CR19] Mehta K (2008). Trends in Radiation Sterilization of Health Care Products. Int at Energy Agency.

[CR20] Mourad R, Helaly F, Darwesh O, El SS (2019). Antimicrobial and physicomechanical natures of silver nanoparticles incorporated into silicone-hydrogel films. Contact Lens Anterior Eye.

[CR21] Rashed A, El-Katatny M, Hetta A, Hashem Z (2020). Validation of moist and dry heat processes used for sterilization and depyrogenation during ampoules manufacturing. J Adv Biomed Pharm Sci.

[CR22] Sage E, Shikazono N (2017). Radiation-induced clustered DNA lesions: Repair and mutagenesis. Free Radic Biol Med.

[CR23] Salmén L, Stevanic JS (2018). Effect of drying conditions on cellulose microfibril aggregation and “hornification”. Cellulose.

[CR24] Silindir M, Özer AY (2009). Sterilization methods and the comparison of E-beam sterilization with gamma radiation sterilization. Fabad J Pharm Sci.

[CR25] Singh R, Singh D, Singh A (2016). Radiation sterilization of tissue allografts: A review. World J Radiol.

[CR26] Vieira FR, Pecchia JA (2018). An Exploration into the Bacterial Community under Different Pasteurization Conditions during Substrate Preparation (Composting–Phase II) for Agaricus bisporus Cultivation. Microb Ecol.

[CR27] William RA, Weber DJ (2008) Guideline for disinfection and sterilization in healthcare facilities

[CR28] Xiang Y, Song Q, Gu W (2020). Decontamination of surgical face masks and N95 respirators by dry heat pasteurization for one hour at 70°C. Am J Infect Control.

